# Gait Speed rather than Dynapenia Is a Simple Indicator for Complex Care Needs: A Cross-sectional Study Using Minimum Data Set

**DOI:** 10.1038/s41598-017-08791-4

**Published:** 2017-08-21

**Authors:** Tzu-Ya Huang, Chih-Kuang Liang, Hsiu-Chu Shen, Hon-I Chen, Mei-Chen Liao, Ming-Yueh Chou, Yu-Te Lin, Liang-Kung Chen

**Affiliations:** 1 Center for Geriatrics and Gerontology, Kaohsiung Veterans General Hospital, Kaohsiung, Taiwan; 2 Department of Family Medicine, Kaohsiung Veterans General Hospital, Kaohsiung, Taiwan; 3 Division of Neurology, Department of Medicine, Kaohsiung Veterans General Hospital, Kaohsiung, Taiwan; 4 Aging and Health Research Center, National Yang Ming University, Taipei, Taiwan; 5 Center for Geriatrics and Gerontology, Taipei Veterans General Hospital, Taipei, Taiwan; 6 Institute of Environmental and Occupational Health Sciences, School of Medicine, National Yang-Ming University, Taipei, Taiwan

## Abstract

The impact of dynapenia on the complexity of care for residents of long-term care facilities (LTCF) remains unclear. The present study evaluated associations between dynapenia, care problems and care complexity in 504 residents of Veterans Care Homes (VCHs) in Taiwan. Subjects with dynapenia, defined as low muscle strength (handgrip strength <26 kg), were older adults with lower body mass index (BMI), slow gait speed, and higher numbers of Resident Assessment Protocol (RAP) triggers. After adjusting for age, education, BMI, and Charlson’s comorbidity index (CCI), only age, education, BMI and gait speed were independently associated with higher numbers of RAP triggers, but *not* dynapenia or handgrip strength (kg). Dividing subjects into groups based on quartiles of gait speed, those with gait speed ≤0.803 m/s were significantly associated with higher complexity of care needs (defined as ≥4 RAP triggers) compared to the reference group (gait speed >1 m/s). Significantly slow gait speed was associated with RAP triggers, including cognitive loss, poor communication ability, rehabilitation needs, urinary incontinence, depressed mood, falls, pressure ulcers, and use of psychotropic drugs. In conclusion, slow gait speed rather than dynapenia is a simple indicator for higher complexity of care needs of older male LTCF residents.

## Introduction

Advanced age and aging is characterized by complex interrelationships between functional decline, multimorbidity, geriatric syndromes and existing disability. The synergistic effects of multimorbidity and geriatric conditions may, in turn, result in greater adverse impacts to the health status of older adults^1, 2^. The adverse effects of geriatric syndromes such as falls, disability, functional decline, cognitive impairment and mortality on older adults have been reported extensively^3–6^. Over two-thirds of older patients present with geriatric syndromes before being admitted to acute-care hospitals, and many of them continue to acquire new geriatric syndromes during hospitalization^7^. People with multimorbidity and geriatric conditions usually receive more intensive and complex care plans. In the United States, for example, these patients account for more than 80% of Medicare expenditure^8^. In 2011, the National Institute on Aging recommended that, to improve quality of care, older adults with multiple chronic conditions should complete a brief initial composite measure, including general health, pain, fatigue, and physical health, mental health and social role function, along with gait speed measurement^9^. It is reasonable then that early identification of complex care needs through the evaluation of composite measures among residents of long-term care facilities (LTCF) may facilitate the development of comprehensive care plans.

Sarcopenia and/or dynapenia are reported to be more prevalent among LTCF residents than among community-dwelling older adults^10, 11^. Sarcopenia was first described by Rosenberg *et al*.^12^ and is characterized by an age-related loss of skeletal muscle mass. However, longitudinal study of associations between muscle mass and functional decline^13^ and early death^14, 15^ showed that the contribution of lower muscle mass on certain health outcomes may be caused partially by low muscle strength. The concept of “dynapenia” proposed by Clark *et al*.^16^ in 2010 described the age-associated loss of muscle strength, which increased risk of functional decline and frailty for older adults. In 2010, the European Working Group on Sarcopenia for Older People (EWGSOP) also defined sarcopenia as the presence of low muscle mass in addition to low handgrip strength and/or slow gait speed, combining measurements of muscle quantity and quality^17^. EWGSOP also used lower handgrip strength as the operational definition of dynapenia^18, 19^, allowing it to become a simple test for evaluating LTCF residents without measuring muscle mass. Although sarcopenia and/or dynapenia are reported to be associated with poor health outcomes^20–23^, the evidence is still inconclusive, and it remains unclear whether dynapenia can effectively represent complexity of care among LTCF residents. Therefore, the main aim of this study was to evaluate the association between dynapenia, care problems and care complexity, to ultimately facilitate the care planning process in LTCFs.

## Methods

### Study design

This study was part of the Longitudinal Older Veterans (LOVE) Study, which was supported by the Taiwanese Veterans Affairs Commission. In the LOVE study, residents of Veterans Care Homes (VCHs) were invited to participate in the study. After the study purpose and the procedures were explained to the residents, those who volunteered to participate were enrolled. All participants provided signed informed consent.

The VCH system is similar to the assisted living and retirement communities in the United States. The Minimum Data Set (MDS Nursing Home 2.1, Chinese version) is a federally mandated process for the comprehensive clinical assessment of LTCF residents’ functional status^24–29^. The MDS was systematically implemented in the VCHs in Taiwan for the purpose of conducting research on resident-related health issues in the long-term care setting.

### Study subjects

In this study, data of subjects living in two VCHs were combined for analysis, including those from Banciao VCH in 2006 and Gansan VCH in 2011. All included subjects were aged 75 years and older and all were male. Subjects with the following conditions were excluded: (1) unable to walk with or without a walking aid, (2) unable to communicate with research nurses, (3) unable or unwilling to provide informed consent, and (4) having a confirmed diagnosis of moderate or advanced dementia. Well-trained research nurses interviewed all participants to collect demographic characteristics, including age, educational level, body mass index (BMI; kg/m^2^), and to complete assessments of the MDS Resident Assessment Protocol (RAP). Multimorbidity was determined using the Charlson’s Comorbidity Index (CCI)^30^. The study protocol was reviewed and approved by the Institutional Review Boards of National Yang-Ming University and Kaohsiung Veterans General Hospital. All the methods described were performed in accordance with the approved guidelines.

### Complexity of care

In this study, complexity of care was evaluated using the MDS RAP triggers, which are specific items defined by the MDS for comprehensive evaluation of residents’ functional capabilities, including delirium, cognitive loss, visual function, communication, rehabilitation needs, urinary incontinence, psychosocial well-being, depressed mood, behavioral symptoms, activities, falls, nutritional status, tube feeding, dehydration, dental care, pressure ulcers, psychotropic drug use and physical restraints. The RAP triggers are measured individually and totally (sum of RAP triggers). Residents’ responses for one or a combination of MDS items helps to identify their needs for specific care problems and are linked to the programmed intervention care plans provided by on-site healthcare professionals. In this study, one RAP trigger (tube feeding) was excluded for analysis because none of the subjects was undergoing tube feeding. All subjects were classified into two groups: those with ≥4 RAP triggers were defined as having high complexity of care needs; those with <4 RAP triggers were defined as having lower complexity of care needs, as previously described^24^. Subjects with lower complexity of care needs were significantly more likely to survive the study period^24^.

### Muscle strength and gait speed measurements

Muscle strength was measured by handgrip strength using a digital dynamometer (TTM-YD, Tokyo, Japan), and the best measurement was recorded based on three trials for the dominant hand, as previously described^30^. All participants also underwent a timed 6-meter walking test at their usual walking speed with a static start throughout a 6-meter distance without deceleration^31, 32^. A research nurse used a stop watch (HS-70 W, Casio Computer, Tokyo, Japan) to record time for the 6-meter walk (seconds). Following the test, time for the 6-meter walk was transformed into gait speed (meters/second). The test allowed the subjects to start with a cane or a walker as needed.

### Definition of dynapenia

Currently, a universal operational definition of dynapenia is still lacking. In this study, dynapenia was defined based on EWGSOP criteria, which uses handgrip strength only^18, 19^. The cut-off point for low muscle strength as defined by dynapenia was handgrip strength <26 kg based on the Asian Working Group for Sarcopenia (AWGS) definition^31^.

### Statistical analysis

In this study, all continuous variables are presented as mean ± standard deviation (SD), and categorical data are presented as numbers (percentage). Student’s t-test was used for comparison of continuous variables between groups, and χ2-test or Fisher’s Exact test were used for comparisons between categorical data when appropriate. Associations between dynapenia and the sum of RAP triggers were evaluated by using multiple linear regression models after adjusting for other covariates, including age, education, BMI and CCI in Model 1, and adjusting for gait speed in Model 2 in addition to the covariates in Model 1. To investigate the influence of handgrip strength itself, we replaced dynapenia with handgrip strength (kg) and also adjusted for all covariates listed above in Model 3. Multivariate logistic regression analysis was used to investigate the risk of higher complexity of care needs (defined as ≥4 RAP triggers), as previously described^24^. All subjects were categorized into four groups according to the quartiles of gait speed to evaluate the associations between gait speed groups and complexity of care needs. For all tests, a two-tailed P-value of <0.05 was considered statistically significant.

## Results

Overall, the data of 504 older adult male residents (mean age 82.6 ± 4.7 years) of Veterans Care Homes (VCHs) in Taiwan were retrieved for analysis. Table 1 summarizes subjects demographic and anthropometric characteristics. Subjects’ mean CCI, gait speed and mean sum of RAP triggers were 0.64 ± 0.83, 0.794 ± 0.324 m/s, and 4.3 ± 2.1, respectively. Univariate analysis showed that residents with dynapenia were older than subjects without dynapenia (82.9 ± 4.9 vs. 82.0 ± 4.3 years, p < 0.001), and also had lower body mass index (BMI) (23.1 ± 3.5 vs. 23.8 ± 3.2 kg/m^2^, p = 0.042), slower gait speed (0.729 ± 0.314 vs. 0.916 ± 0.307 m/s, P < 0.001), lower handgrip strength (18.7 ± 4.6 vs. 29.8 ± 3.4 kg, p < 0.001), and a higher sum of RAP triggers (4.5 ± 2.3 vs. 3.8 ± 1.7, p < 0.001) than subjects without dynapenia (Table 1).

**Table 1:** Subjects’ demographic and anthropometric characteristics with or without dynapenia*.

	Total N = 504% or mean ± SD	Dynapenia	Non-Dynapenia	P value
		N = 330% or mean ± SD	N = 174% or mean ± SD	
Age^†^	82.6 ± 4.7	82.9 ± 4.9	82.0 ± 4.3	<0.001
Educational level^‡^				0.922
No formal education	268 (53.2%)	176 (53.3%)	92 (52.9%)	
Higher than Primary school	236 (46.8%)	154 (46.7%)	82 (47.1%)	
Body mass index (kg/m2)^†^	23.3 ± 3.4	23.1 ± 3.5	23.8 ± 3.2	0.042
Charlson’s comorbidity index ^†^	0.64 ± 0.83	0.69 ± 0.87	0.55 ± 0.75	0.073
Gait speed (m/s)^†^	0.794 ± 0.324	0.729 ± 0.314	0.916 ± 0.307	<0.001
Handgrip stretch (kg)^†^	22.5 ± 6.8	18.7 ± 4.6	29.8 ± 3.4	<0.001
Sum of RAP triggers^†^	4.3 ± 2.1	4.5 ± 2.3	3.8 ± 1.7	<0.001

Values presented as ^†^mean ± standard deviation; ^‡^number (%).

*Defined by EWGSOP criteria (European Working Group on Sarcopenia for Older People, 2010^17^).

DLMS: Based on Handgrip strength <26 kg.

RAP: Resident Assessment Protocol.

In Table 2, results of multiple linear regression analysis show that the presence of dynapenia is strongly associated with higher numbers of RAP triggers after adjusting for covariates of age, education, BMI and CCI in Model 1 (standardized co-efficient of dynapenia: 0.124, p = 0.005), but the association disappears after adding gait speed as a covariate in Model 2 (standardized co-efficient for dynapenia: −0.060, p = 0.168). Otherwise, slow gait speed (standardized co-efficient for gait speed: −0.270, p < 0.001) and older age (standardized co-efficient: 0.146, p = 0.001), lower educational level (−0.089, p = 0.036), and lower BMI (−0.100, p = 0.019) were independently associated with higher numbers of RAP triggers. In Model 3, handgrip strength (kg) was considered a dependent variable to evaluate associations with higher numbers of RAP triggers. However, no significant associations were found between handgrip strength and higher numbers of RAP triggers (standardized co-efficient of handgrip strength: −0.070, p = 0.128), although the association between gait speed and a higher number of RAP triggers was still statistically significant (standardized co-efficient of gait speed: −0.260, p < 0.001) (Table 2).

**Table 2:** Multiple linear regression analysis of risk factors for higher complexity of care needs* according to RAP triggers.

	Model 1			Model 2			Model 3		
	β (standardized co- efficient)	t	p	β (standardized co- efficient)	t	p	β (standardized co- efficient)	t	p
Age	0.215	4.826	<0.001	0.146	3.277	0.001	0.144	3.229	0.001
Education(Yes)	−0.094	−2.140	0.033	−0.089	−2.102	0.036	−0.087	−2.066	0.039
CCI	0.077	1.759	0.079	0.038	0.896	0.371	0.038	0.878	0.380
BMI	−0.092	−2.093	0.037	−0.100	−2.359	0.019	−0.098	−2.301	0.022
Dynapenia(Yes)	0.124	2.819	0.005	−0.060	1.380	0.168			
Gait speed(m/s)	—	—	—	−0.270	−5.958	<0.001	−0.260	−5.505	<0.001
Handgrip strength(kg)	—	—	—				−0.070	1.525	0.128

^*^Higher complexity of care needs defined as ≥4 RAP triggers.

CCI: Charlson’s comorbidity index; BMI: body mass index; RAP: Resident assessment protocol.

Dynapenia: Based on Handgrip strength <26kg.

Model 1: Adjusted for Age, Educational level, CCI, BMI, and Dynapenia, R^2^ = 0.088.

Model 2: Adjusted for Age, Educational level, CCI, BMI, Dynapenia and Gait speed, R^2^ = 0.160.

Model 3: Adjusted for Age, Educational level, CCI, BMI, Handgrip strength (kg) and Gait speed, R^2^ = 0.149.

Since slow gait speed was highly associated with higher numbers of RAP triggers, subjects were divided into four groups based on quartiles of gait speed in order to further evaluate associations between slow gait speed (Q 1: gait speed >1.000 m/s; Q 2: gait speed 0.804–1.000 m/s; Q 3: gait speed 0.546–0.803 m/s; Q 4: gait speed < 0.546 m/s) and higher complexity of care needs (defined as ≥ 4 RAP triggers). Compared to Q1, the higher gait speeds in Q3 and Q4 groups were significantly associated with higher complexity of care needs after adjusting for all covariates, except for Q2, as shown in Table 3 (Q2 group adjusted OR(aOR):1.627, CI: 0.946–2.800, P = 0.079; Q3 group sOR: 2.482, CI: 1.389–4.435, P = 0.002; Q4 group sOR: 4.224, CI: 2.235–7.981, P < 0.001). Dynapenia was not independently or significantly associated with higher complex care needs in multivariate logistic regression analysis (aOR:0.951, CI: 0.635–1.425, P = 0.808) (data not shown) (Table 3).

**Table 3:** Multivariate logistic regression for independent associations between gait speed quartiles and higher complexity of care needs.

Gait Speed Group	Unadjusted			Adjusted		
	OR	95% CI	P value	OR	95% CI	P value
Q 1	Reference	Reference		Reference	Reference	
Q 2	1.763	1.047 – 2.969	0.033	1.627	0.946–2.800	0.079
Q 3	2.696	1.545–4.705	<0.001	2.482	1.389–4.435	0.002
Q 4	4.715	2.643–8.411	<0.001	4.224	2.235–7.981	<0.001

Adjusting for age, education, BMI, CCI, Dynapenia.

CCI: Charlson’s comorbidity index; BMI: body mass index; RAP: Resident assessment protocol.

Dynapenia: Based on Handgrip strength <26 kg.

Higher complexity of care needs: defined as ≥4 RAP triggers.

Q 1: gait speed >1.000.

Q 2: gait speed 0.804~1.000.

Q 3: gait speed 0.546~0.803.

Q 4: gait speed <0.546.

Table 4 compares mean gait speeds between residents with individual RAP triggers. Slow gait speed was significantly associated with individual RAP triggers, including cognitive loss (0.695 ± 0.302 vs. 0.822 ± 0.325 m/s, P < 0.001), poor communication ability (0.709 ± 0.297 vs. 0.843 ± 0.329 m/s, P < 0.001), rehabilitation needs (0.751 ± 0.356 vs. 0.819 ± 0.301 m/s, P = 0.028), urinary incontinence (0.569 ± 0.311 vs. 0.814 ± 0.318 m/s, P < 0.001), depressed mood (0.686 ± 0.323 vs. 0.822 ± 0.318 m/s, P < 0.001), falls (0.622 ± 0.294 vs. 0.818 ± 0.321, P < 0.001), pressure ulcers (0.545 ± 0.316 vs. 0.808 ± 0.318 m/s, P < 0.001), and use of psychotropic drugs (0.667 ± 0.299 vs. 0.861 ± 0.317 m/s, P < 0.001).

**Table 4:** Comparisons of mean gait speed between residents with individual RAP triggers.

	Gait speed (meter/s)				
	RAP trigger (+)		RAP trigger (−)		*P* value
	N	Mean ± SD	N	Mean ± SD	
1. Delirium	32	0.693 ± 0.313	472	0.799 ± 0.323	0.073
2. Cognitive loss	113	0.695 ± 0.302	391	0.822 ± 0.325	<0.001
3. Visual function	195	0.772 ± 0.336	309	0.810 ± 0.315	0.228
4. Communication	185	0.709 ± 0.297	319	0.843 ± 0.329	<0.001
5. Rehabilitation needs	188	0.751 ± 0.356	316	0.819 ± 0.301	0.028
6. Urinary incontinence	42	0.569 ± 0.311	462	0.814 ± 0.318	<0.001
7. Psychosocial well-being	332	0.777 ± 0.314	172	0.825 ± 0.341	0.116
8. Depressed mood	106	0.686 ± 0.323	398	0.822 ± 0.318	<0.001
9. Behavioral symptoms	2	0.875 ± 0.177	502	0.793 ± 0.324	0.722
10. Activities	228	0.773 ± 0.348	276	0.810 ± 0.302	0.206
11. Falls	63	0.622 ± 0.294	441	0.818 ± 0.321	<0.001
12. Nutritional status	14	0.672 ± 0.333	490	0.797 ± 0.323	0.153
13. Tube Feeding	0	—	0	—	—
14. Dehydration	8	0.614 ± 0.356	496	0.797 ± 0.323	0.113
15. Dental care	412	0.796 ± 0.326	92	0.784 ± 0.316	0.765
16. Pressure ulcers	27	0.545 ± 0.316	477	0.808 ± 0.318	<0.001
17. Psychotropic drug use	175	0.667 ± 0.299	329	0.861 ± 0.317	<0.001
18. Physical Restraints	1	0.286	503	0.795 ± 0.323	0.117

Mean gait speed for total 504 subjects: 0.794 ± 0.324 meter/s.

RAP: Resident assessment protocol.

## Discussion

To the best of our knowledge, this study is the first to evaluate associations between dynapenia and the complex care needs of LTCF residents using Minimum Data Set (MDS) assessments. In this study, the mean number of MDS RAP triggers of study subjects was around 4 and the mean gait speed (0.794 ± 0.324 m/s) was slower than the diagnostic criteria for sarcopenia recommended by EWGSOP and AWGS^17, 31^. Due to the older age of study participants (all aged 75 years and older), slow gait speed of these subjects was compatible with previous findings. In this study, we found that slow gait speed, rather than dynapenia or handgrip strength, was significantly associated with higher complexity of care needs. Residents with RAP triggers of cognitive loss, poor communication ability, rehabilitation needs, urinary incontinence, depressed mood, falls, pressure ulcers and use of psychotropic drugs had significantly slower gait speed.

Despite the lack of a universal working definition for dynapenia, handgrip strength has been used to describe the age-related loss of muscle strength. Also, even though some previous studies identified associations between dynapenia and existing cognitive dysfunction, future morbidity, hospitalizations, disability and mortality^20, 23^, recent evidence from EWGSOP could not confirm the relationship between dynapenia and lower handgrip and disability^18, 19^. In the present study, no significant associations were found between dynapenia and higher complex care needs for LTCF residents. In addition, the impact of low handgrip strength on health outcomes of older adults, including functional decline, disability and mortality, was not demonstrated in several investigations^33–36^, but results of other studies still indicate that no association is found between handgrip strength and all-cause mortality, and no certification for future long-term care^37, 38^. Even with continued study, predictive factors for complexity of care for LTCF residents remains unclear, and evaluations of associations between care needs, utilization of care and handgrip strength are lacking.

Results of the present study showed a nonsignificant association between low handgrip strength and complex care needs. When the handgrip cut-off point was reset using the lowest tertiles of handgrip strength (<19.33 kg), the results remained the same as using the cut-off point of 26 kg for dynapenia (data not shown). Compared to low handgrip strength, slow gait speed has been recognized as a better indicator of cumulative deficits over multiple physiologic systems^39^, which may also be more effective in defining older adult subjects with higher health risk profiles. Walking is a complex task that requires sophisticated coordination of multiple body systems^39^. In the present study, we found that slow gait speed is significantly associated with the presence of multiple geriatric health problems as represented by RAP triggers (Pearson correlation coefficient −0.318; p < 0.001; data not shown). A previous study showed that gait speed measurement alone was usually sufficient to detect five geriatric syndromes (urinary incontinence, falls, underweight, presence of depression and functional decline) among community-dwelling women compared to a more comprehensive test battery^40^. Similarly, results of the present study with all male participants also showed that a simple gait speed measurement may effectively identify risk of higher complexity of care needs of LTCF residents. Moreover, a significant association between slow gait speed and higher complexity of care needs was identified by using the cut-off point below 0.803 m/s (Q3: OR 2.482, 95% CI 1.389–4.435, p = 0.002) (Q3), which was comparable to the definition of slowness in the sarcopenia diagnosis according to EWGSOP and AWGS criteria^17, 31^. Gait speed slower than 0.546 m/s (Q4) was highly associated with high complexity of care needs (OR 4.224, 95% CI 2.235–7.981, p < 0.001). In another previous study, a gait speed of ≤0.8 m/s among men aged 75 years and older was also associated with various geriatric syndromes in addition to adverse health outcomes^32^, indicating that gait speed may be a simple and effective screening tool for risk of higher complexity of care needs. The gait speed cut-off point (≤0.8 m/s) in that male-predominant study sample differs from the gait speed of 1.0 m/s suggested as an ideal cut-off point for adverse health outcomes in women aged 75 years and older^40^. The gender difference in the cut-off point for gait speed of older adults warrants further research efforts^41^.

In the present study, apart from slow gait speed, higher complex care needs were significantly associated with older age, low educational levels and lower BMI, but not with multimorbidity or lower handgrip strength. Older age was a strong risk factor for multimorbidity and geriatric syndromes, whereas lower BMI may be an indicator for undernutrition. Both older age and low BMI were associated with mortality and hospitalizations for older Chinese adults living at home or institutions^42^. The synergistic relationship between aging and multimorbidity significantly increased the risk of adverse outcomes among older people, including disability and functional decline, poorer quality of life, and higher health care costs^1^. However, multimorbidity in older adults represented a static health state that may not completely represent the current health status and care needs at personal or institutional levels^23^. A previous study conducted in the geriatric rehabilitation setting suggests that the effect of multimorbidity on post-hospitalization outcomes is eventually mediated by functional status^43^. In other words, functional status may outweigh multimorbidity in determining the overall health status of older adults.

Gait speed at an individual’s usual pace has become the “sixth vital sign” or a “functional vital signs,”^44^ and it has been identified as a risk factor for disability, cognitive impairment, and falls^20, 45^. Previous studies have identified that slow gait speed is associated with greater hearing loss and poorer objective physical functioning among older adults^46, 47^. These findings may be explained by the presence of micro- cerebrovascular disease, pathology of both cochlear and vestibular sense organs, and hearing impairment-related reductions in social, physical and cognitive activities^46, 47^. Since communication difficulties are highly associated with cognitive impairment and hearing impairment, the association between communication difficulties and slow gait speed in this study may be secondary. The association between urinary incontinence and slow gait speed was also reported in a study focused on women only^48^. However, urinary incontinence may be an important contributing factor for slow gait speed, and vice versa^49^. The common pathoetiology of urinary incontinence and slow gait speed may be a result of cerebral white matter hyperintensities since the severity or distribution of white matter lesions derived from cerebral hypoperfusion may lead to issues of mobility, voiding and cognitive dysfunction occurring at the same time^50^. Meanwhile, the presence of cognitive impairment, depressive symptoms and sarcopenia may slow the gait speed of older adults^51, 52^, and slow walking speed and falls occurred more frequently in subjects with cognitive impairment and depression^53^. Such findings suggest that, the underlying pathophysiology of individual RAP triggers as related to slowness may vary greatly between individuals.

### Limitations

This study has several limitations. First, the demographic characteristics of participants represented a homogeneous sample, which may limit the extrapolation of results to the general population. Second, some residents refused to participate in this study, mainly because they considered themselves to be healthier than other residents and lacked the time for periodical assessments or were unable to walk due to advanced frailty status. Therefore, selection bias may still exist, limiting the interpretation and application of study results only to included subjects able to walk. Third, residents with moderate-to-severe dementia and other major illnesses were not included in this study, so the results may not be applied to subjects with mental illnesses. Fourth, information on subjects’ physical activity and body composition were not entered into analysis and the possible influence of physical activity and body composition on the results of other parameters could not be evaluated. Fifth, the cross-sectional observational design also limits the possibility of identifying causal relationships between dynapenia and the complexity of care needs.

## Conclusion

Results of the present study show that slow gait speed is significantly associated with the presence of multiple complex care needs in LTCF residents and, as such, gait speed is able to quickly and easily indicate higher complexity of care needs in older male residents. A simple and effective 6-meter walking test is an ideal instrument by which to define gait speed and identify LTCF male subjects with multiple health problems that require care. Identifying LTCF residents with complex care needs is essential to help improve the quality of care and outcomes of long-term care residents. Further prospective study is needed to evaluate the possibility of reducing complex care needs for LTCF residents by applying intervention strategies to improve gait speed.
